# A Plea for the More General Use of Alkaloids

**Published:** 1891-08

**Authors:** Vard H. Hulen

**Affiliations:** Galveston


DANIEL'S

### Texas Medical Journal.

A Representative Organ of the Medical Profession, and an Exponent of Rational Medicine; devoted to the Organization, Advancement and Elevation of the Profession in Texas.

PUBLISHED MONTHLY.—SUBSCRIPTION $2.00 A YEAR.

Vol. VII. AUSTIN, AUGUST, 1891. No. 2.

### Original Articles.

CONTRIBUTED EXCLUSIVELY TO THIS JOURNAL.

*The Articles in this Department are accepted and published with the understanding that we are not responsible for, nor do we indorse the views and opinions of the writers by so doing.*

---

For Daniel's Texas Medical Journal.

### A PLEA FOR THE MORE GENERAL USE OF ALKALOIDS.

BY VARD H. HULEN, M. D., GALVESTON.

[Read before Galveston County Medical Club, July 12, 1891.]

EACH and every member of the professions of medicine and pharmacy knows only too well that the various preparations of vegetable drugs are unscientific and unsatisfactory as now manufactured according to the rules of the pharmacopœia.

Our therapeutical knowledge, although far from perfect, is sufficiently advanced to demand more accurate remedial agents. It is possible for the life of the patient to depend greatly on the purity and correct dosage of the medicine. In a case of post partum hemorrhage, would the accoucheur have implicit confidence in any of the Galenical preparations of ergot?

The usefulness of the physician is destroyed, to a considerable extent, by the unreliability of medicines as found on the shelves of our drug stores to-day. Personally, I have not an unlimited

"faith" in drugs; but I do believe that with pure medicines and intelligent administration suffering can be alleviated, recovery hastened, and lives saved.

Our therapeutics are based on the hypothesis that drugs are uniform in quality and composition. This is a delusion and a snare. We all know that the strength varies greatly, depending on the locality in which the specimen is grown, the age of the plant, the season the drug is gathered, the time between gathering and manufacturing, the time between manufacturing and dispensing, and the difference between the methods of manufacturers and dispensers. In the purchase of crude drugs, the quality is largely judged by the physical indications, and appearances are as deceptive in drugs as in people.

Dr. H. H. Rusby, of Columbia College, New York, assayed twenty-three specimens of aconite root, and found the maximum strength twice that of the minimum; six specimens of cinchona calisaya, in which the maximum was almost two and one-half times that of the minimum; ten specimens of colchicum seed, the maximum being more than two and one-half times as strong as the minimum; eight specimens of ipecac, in the strongest specimen there was more than twice the amount of emetin found in the weakest; nine specimens of nux vomica, the amount of alkaloid in the maximum being over four times as great as that in the minimum; and various other drugs with similar results.

Dr. Willis G. Tucker, Analyst of the State Board of Health of New York, examined sixty-eight samples of drugs collected last May in five cities in his State. Of this number, thirty-three, almost half, were deficient in quality, hence unreliable.

It has been computed that the chances of getting drugs of *good* quality on prescriptions are less than 44%; fair, 17%; inferior, 26%; not as called for, 12%; and excessive strength, 1%. This includes toxic and narcotic drugs. Just think of it!

When it is remembered that fluid extracts, tinctures, wines, decoctions, etc., are manufactured so that a definite quantity of the preparation represents a certain percentage of *weight* of crude drug, regardless of the amount of active principle, it is readily seen how unreliable are the Galenical preparations, even when freshly prepared. Then when we consider the length of time these preparations may have been standing on the shelves of the drug stores, undergoing chemical and other changes, before using, it is surprising that we get any positive result at all. It is not an unheard of thing for a physician to be disappointed by a remedy, when on changing the preparation, or the druggist, the expected result is readily obtained, showing conclusively the worthlessness of the first article. It is for this reason that physicians "specify" the manufacturer, and direct their patients to the most reliable druggists.

The *St. Louis Weekly Medical Review*, truly says, that "Medical practice upon a basis which allows the existence, in a row of fifty consecutive drug stores, forty different strengths of the same preparations, ranging from 25 to 150%, is not much better than semi-barbarous."

If we should tell a business man, a dealer in percentages, that the medicines we were giving him varied in strength from 25 to 150%, he would certainly dispense with our medicines at once, and our services as well, and denounce us as impostors.

If, in a critical case, we have to deal with an uncertain diagnosis, an indifferent druggist, an unreliable preparation, an inaccurate dosage and an ignorant nurse, what chance is there for the patient? And this very condition of affairs is not impossible in this, the boasted nineteenth, century.

Life and death and humanity, as well as the reputation of the medical profession, are staked on such untrustworthy preparations, yet how little are we exercised over this deplorable state of our therapeutical agents.

Neither the wholesale nor retail druggists are responsible for this, though they can assist greatly in the improvement of preparations. Among pharmacists are as honest, upright, hard-working, painstaking and intelligent men as can be found in any business. But the fact remains the same that modern pharmacy does not satisfy the demands of modern therapeutics. It is no great wonder that practitioners are using more and more of the "ready made" prescriptions of manufacturers.

Will you permit me to digress long enough to protest against this practice?

A few of these proprietary medicines are useful, but we should use none of them when the composition is a secret one, or when the exact amount of each ingredient is unknown.

A majority of the formulæ, so-called, accompanying these preparations, is unintelligible to doctor and druggist, hence the preparations are secret nostrums, and how can we, as rational physicians, prescribe them? The use of these medicines dwarfs the thoughtfulness of practitioners, as is is not required of him to determine the quality, quantity or dose of his therapeutical agents. He simply writes:

℞ Short & Sweet's Quasiquack Elixir....One bottle.
Signa. A teaspoonful three or four times daily.
N. G., M. D.

And it is an injury to the pharmacist, for all the skill necessary for filling such a prescription is enough to find this particular preparation among so many in stock of the same character, and write the label. Or if he does not have this special brand, and what druggist can keep them all, he must go to the extra trouble and expense of "sending out" for it. Can we blame the druggist if he sometimes deplores the lack of originality of the doctors?

But these objections carry little weight as compared with the fact that prescribing these preparations is an injustice to the patient, for such mixtures are not so nicely adjusted to his condition in composition and dose as a carefully considered prescription would be. And it is for this, as well as a diagnosis, the physician is consulted.

But to return to my subject. I do not advocate the use of active principles, neither alkaloids nor glucosides, to the exclusion of other preparations. We may desire the effect of the crude drug, and not the alkaloid alone; for instance, opium may be indicated, and not morphine. When crude preparations are advisable, the "normal liquids" of Parke, Davis & Co. are strongly recommended. They are manufactured so that a certain quantity of the normal liquid represents a definite *strength* of active principles, and are assayed to this standard, regardless of the amount of crude drug necessary, and it is readily seen how much more reliable these liquids are than the pharmacopœial preparations.

Also, I certainly would not exclude other mineral and vegetable drugs which have no alkaloids or active principles, such as the salts of potassium and sodium, magnesium and ammonium, chloral, camphor and chloroform, arsenic, iron and mercury, alcohol, the oils and acids, and all such useful and necessary medicines.

It is encouraging to know that many of the alkaloids are frequently used now, as atropine, apamorphine, antifebrine, antipyrine, cocoaine, caffeine, codeine, morphine, quinine, pilocarpine and strychnine. Of the glucosides used are, elaterine, picrotoxin, salicin, and santonin.

Why, in addition to these, should we not use aconitine, aloine, brucine, cannabine; cicutine, cubebine, digitaline, emetine, ergotine, gelsemine, hyoscyamine, jalapine, kousseine, pelleterine, physostigmine, quasseine, strophautine, and veratrine?

The advantages I claim for alkaloids over Galenical medications, are:

*First*, and most important, a greater uniformity and reliability of preparations.

*Second*, increased accuracy of doses.

*Third*, the avoidance of incompatibility in prescriptions.

*Fourth*, a more distinct and limited effect of medicines; for in giving alkaloids we get only the action of the one agent, and not the effect of from one to a half dozen other ingredients, as with Galenical preparations.

*Fifth*, a greatly improved method in the administration of remedies to the patient. Drops and spoonfuls are the usual measures for ordinary preparations: drops vary in number from 44 to 250 to the fluid drachm, and the sizes of spoons vary more than 100%, to say nothing of the deficient filling and overflowing of spoons and the spilling of medicines in administration.

*Sixth*, a more concise method of treatment of disease. The prescriber must have a clear idea of what he wishes to accomplish. The action of each alkaloid is definite, and the indications are plain. "Shotgun" prescriptions with alkaloids, are out of the question. The "hit or miss" plan, in which the right thing is usually missed and the wrong thing hit, will not do.

If I have settled beyond a doubt that alkaloids are superior in *action* to the Galenical preparations, then I pass to the

*Seventh*, and last advantage I claim for them, this is a pleasant form for administration. The alkaloidal granules of the Metric Granule Co., Ch. Chauteaud & Co., and others, are quite small, are readily placed in a child's mouth, and swallowed often without a question.

Can you imagine a more ridiculous scene than that presented by a child and its attendants on the arrival of the hour at which a dose of disgusting medicine must be given? The hero is held in the arms of its grandmother, for the father usually finds it convenient to be elsewhere on such a momentous occasion. There is vigorous kicking and squalling on the part of the child as the mother holds his nose and awaits an opportunity to pour down its unwilling throat the vile mixture. For the sake of safety, the spoon is held high in air, but some of the contents is spilt at every kick of the young rebel. The farce ends by the bulk of the medicine going down the *outside* of the victim's neck, only enough reaches the inside to give him the benefit of its nauseating taste, which causes the little patient to pass new and firmer resolutions to be more successful in the succeeding attacks.

Often the excitement attending such exhibitions does more harm than the medicine does good. Many times mothers love their children, "not wisely, but too well" to thus punish them, and in consequence let the little sufferers go without the medicine.

A great many sick children, some of them pretty *old* children, too, dread the sight of a doctor, because they know his visits mean vile smelling and worse tasting medicine. For this very reason, people sometimes postpone sending for a "medicine man" until the diseased condition is much more difficult to deal with than it would have been at first. And it is not pleasant or complimentary to the doctor, on entering the sick chamber, to be greeted by the little patient with "weeping, and wailing, and gnashing of teeth," as if his Satanic majesty had called to claim his own.

It has been my misfortune to make second visits to both adults and children and find the medicine previously ordered carefully and surely put away after the first dose; the patient no better, time lost, the physician's endeavors have been in vain and unappreciated, and every body disgusted.

Why persist in giving to weak and nervous patients, or any body else, these stomach turning doses, and thus pave the way for irregulars, when we have superior and pleasant remedies at our command?

I am convinced that nine-tenths of the practice of homeopaths is due to just two things, one is that their medicines are palatable, and the other is that their patients are at no additional expense for medicines.

If it is desirable to give more than one kind of the granules at a time, they can easily be enclosed in a small capsule. If for any reason the administration of granules is impracticable, they can readily be dissolved in water. To the physician who carries his own medicine, they are an invaluable acquisition, and every practitioner shuld keep a few of the granules or tablets for immediate use.

The use of the alkaloids is not adopting a new system, or a

"system" of any kind. But it is practicing physic with the same old drugs in a rational way as usual, applying the facts as determined by physiological and clinical researches recorded in the standard works of Wood, Bartholow, Ringer, T. Lauder Brunton, and other original investigators.

We are members of a liberal profession, and as regular physicians pride ourselves on the broad basis of our practice, using anything and everything for the benefit of the sick, regardless of system, ism or pathy. We must follow up every advance, and adopt every improvement, hence my plea for the more general use of alkaloids.

